# All-Cause and Cause-Specific Risk of Emergency Transport Attributable to Temperature: A Nationwide Study: Erratum

**DOI:** 10.1097/01.md.0000504797.68207.2e

**Published:** 2016-10-21

In the article “All-Cause and Cause-Specific Risk of Emergency Transport Attributable to Temperature: A Nationwide Study”,^[1]^ which appeared in Volume 94, Issue 51 of *Medicine*, the editor Roman Leischik's name was originally omitted. The article has since been corrected online.
